# Urinary DNA methylation biomarkers for prediction of prostate cancer upgrading and upstaging

**DOI:** 10.1186/s13148-019-0716-z

**Published:** 2019-08-05

**Authors:** Arnas Bakavicius, Kristina Daniunaite, Kristina Zukauskaite, Marija Barisiene, Sonata Jarmalaite, Feliksas Jankevicius

**Affiliations:** 1 Institute of Clinical Medicine, Faculty of Medicine, Vilnius University, Vilnius, Lithuania; 2 National Cancer Institute, Vilnius, Lithuania; 3 Vilnius University Hospital Santaros Klinikos, Vilnius, Lithuania; 4 Institute of Biosciences, Life Sciences Center, Vilnius University, Vilnius, Lithuania

**Keywords:** Prostate cancer, DNA methylation, Upgrading, Upstaging, Urine

## Abstract

**Background:**

Significant numbers of prostate cancer (PCa) patients experience tumour upstaging and upgrading in surgical specimens that cause serious problems in timely and proper selection of the treatment strategy. This study was aimed at the evaluation of a set of established epigenetic biomarkers as a noninvasive tool for more accurate PCa categorization before radical prostatectomy (RP).

**Methods:**

Quantitative methylation-specific PCR was applied for the methylation analysis of *RARB*, *RASSF1*, and *GSTP1* in 514 preoperatively collected voided or catheterized urine samples from the single-centre cohort of 1056 treatment-naïve PCa patients who underwent RP. The rates of biopsy upgrading and upstaging were analysed in the whole cohort.

**Results:**

Pathological examination of RP specimens revealed Gleason score upgrading in 27.2% and upstaging in 20.3% of the patients with a total misclassification rate of 39.0%. DNA methylation changes in at least one gene were detected in more than 80% of urine samples. Combination of the PSA test with the three-gene methylation analysis in urine was a significant predictor of pathological upstaging and upgrading (*P* < 0.050), however, with limited increase in overall accuracy. The PSA test or each gene alone was not informative enough.

**Conclusions:**

The urinary DNA methylation assay in combination with serum PSA may predict tumour stage or grade migration post-RP aiding in improved individual risk assessment and appropriate treatment selection. Clinical utility of these biomarkers should be proven in larger multi-centre studies.

**Electronic supplementary material:**

The online version of this article (10.1186/s13148-019-0716-z) contains supplementary material, which is available to authorized users.

## Background

Prostate cancer (PCa) treatment selection mostly depends on tumour biopsy-based Gleason score (GS) and clinical tumour stage (cT). Upgrading or upstaging to advanced disease after pathological examination has emerged as a serious issue in PCa diagnostics and is reported in 40–60% of PCa patients [1–4]. The proper treatment of the patients, whose cancer is later upgraded and/or upstaged, might be delayed resulting in irreversible consequences, while overtreatment is a serious concern for men whose biopsies are downgraded. The subsequent upgrading or upstaging of biopsy results has been associated with an increased risk of PCa biochemical recurrence after radical prostatectomy (RP) and other serious clinical consequences [5]. More accurate PCa characterization at diagnosis is critical for the efficient management of the disease; however, current clinical tools are not effective enough to provide an accurate diagnosis following the primary assessment of the disease.

Over the last years, rapid development of genomic technologies and their application for deciphering cancer genome have offered new diagnostic possibilities for PCa patients. Identification of cancer-associated genetic and epigenetic alterations in body fluids, containing molecular information from all tumour foci and reflecting PCa heterogeneity, may provide valuable supplementary data for improved diagnosis and timely prediction of PCa aggressiveness [6–8]. DNA methylation changes in tumour suppressor genes (TSGs) occur early in prostate carcinogenesis and are suggested to be a key element of cancer progression [9]. PCa-specific methylated DNA is easily detectable in liquid biopsy samples, such as urine or blood, and can provide additional information beyond the limitations of standard prostate biopsy [10].

In the present study, we assessed the performance of the established clinical predictors of PCa outcome, and DNA methylation of three TSGs, known to be associated with PCa [6, 11]—retinoic acid receptor β (*RARB*), RAS association domain family member 1 (*RASSF1*), and glutathione *S*-transferase pi 1 (*GSTP1*)—as potential noninvasive biomarkers for more accurate PCa risk assessment.

## Methods

### Patients and samples

Clinical data of 1056 treatment-naïve patients with histologically confirmed PCa (at least 10-core random biopsy sampling) who underwent RP at Vilnius University Hospital Santaros Klinikos between January 2008 and December 2014 were analysed in the study for the estimation of the upgrading and upstaging rates. For the molecular analysis, urine samples were available from 514 of 1056 patients (Additional file 1: Table S1). Previous androgen-deprivation therapy, active surveillance, and history of urothelial carcinoma were considered as exclusion criteria.

Urine specimens were collected using two different clinically applicable techniques: voided urine samples (*N* = 188) were collected after the prostate massage in the morning before the surgery; catheterized urine (*N* = 326) was obtained under general anaesthesia immediately before the surgery. All urine samples were processed according to the standard protocol [6]. A set of data from the catheterized urine samples (253 of 326) has been reported in our previous study [6]. Fresh-frozen tissue samples of 111 prostate tumours and 16 noncancerous prostate tissues (NPT) from RP material were also available for the analysis (Additional file 1: Table S1).

GS was evaluated according to the 2005 Guidelines of International Society of Urological Pathology (ISUP), and ISUP grade groups (GG) were assigned according to ISUP 2014 recommendations [12, 13]. Upgrading was defined as any increase of GG between biopsy (clinical GG; cGG) and RP pathology (pathological GG; pGG), i.e. pGG>cGG, whereas upstaging was confirmed if a patient was pathologically diagnosed with advanced disease (≥ pT3) when clinically unsuspected. Based on GG and tumour stage, all patients were stratified into preoperative and postoperative PCa risk groups (preoperatively—low (cGG 1 and ≤ cT2a), intermediate (cGG 2–3 and/or cT2b), and high (cGG 4–5 and/or ≥ cT2c), and postoperatively—low (pGG 1 and ≤ pT2c), intermediate (pGG 2-3 and ≤ pT2c), and high (pGG 4-5 and/or ≥ pT3a). All clinico-pathological parameters of the study cohorts are summarized in Additional file 1: Table S1.

### DNA extraction and bisulfite conversion

Prostate tissue samples were mechanically homogenized in liquid nitrogen, and 10–30 mg of powder were used for genomic DNA purification according to the standard phenol-chloroform protocol. DNA from urine was purified as described previously [6]. For the methylation analysis, 400 ng of extracted DNA were bisulfite-modified using EZ DNA Methylation^TM^ Kit (Zymo Research, Irvine, CA, USA) according to the manufacturer’s instructions, except that the initial incubation of samples was performed at 42 °C for 15 min.

### DNA methylation analysis

All primers were designed according to the previously published sequences [6]. In tissues, DNA methylation was evaluated by means of qualitative methylation-specific PCR (MSP). The reaction volume (25 μL) consisted of 1x Maxima Hot Start Taq PCR buffer, 2.5 mM of MgCl_2_, 1.6 mM of dNTP mix, 1.25 U of Maxima Hot Start Taq DNA Polymerase (all from Thermo Scientific^TM^, Thermo Fisher Scientific, Vilnius, Lithuania), 1 mM of each primer (Metabion, Martinsried, Germany), and 1 μL of modified DNA. Thermocycling conditions included 37-39 cycles with primer annealing step at 60–62 °C for 45 s. Methylation-positive (in vitro fully methylated leukocyte DNA; MC), methylation-negative (leukocyte DNA from a healthy male donor), and nontemplate controls (NTC) were included in each MSP assay.

In urine samples, DNA methylation was analysed using quantitative MSP. The reaction mix (20 μL) consisted of 1x TaqMan Universal Master Mix II no UNG (Applied Biosystems^TM^, Thermo Fisher Scientific, Carlsbad, USA), 50 nM of hydrolysis probe, 300 nM of each primer, and 1 μL of modified DNA. All experiments were carried out at 95 °C for 10 min followed by 50 cycles of 95 °C for 15 s and 60 °C for 1 min. A run was considered valid when routinely included MCs gave a positive signal and there was no amplification in NTC. A sample was classified as valid if the cycle of quantification (Cq)-value of the endogenous control *ACTB* was < 40 in all three replicates per sample. Methylation level of a particular gene was estimated based on ΔΔCq algorithm and expressed as a percentage of the MC. For the qualitative analysis, samples were dichotomized into methylated and unmethylated considering the 0.1% methylation level as the threshold, which was selected based on the data repeatability and the technical limit of detection, as well as on the consistency with the MSP results of the same samples (not shown).

### Statistical analysis

Statistical analysis was performed with STATISTICA™ v8.0 (StatSoft, Tulsa, USA) and MedCalc® v12.7 software (MedCalc Software, Ostend, Belgium). Two-sided Fisher’s exact test and Mann-Whitney *U* test were used for two-group comparisons of categorical and continuous data, respectively. Methylation levels are provided as means with standard error of mean (SEM). The ability of the biomarkers to distinguish groups was evaluated by performing receiver operating characteristic (ROC) curve analysis and estimating the area under the curve (AUC) values. The test’s performance parameters—sensitivity, specificity, positive predictive value (PPV), and negative predictive value (NPV)—were obtained from the ROC curve analysis and based on the Youden index for the selection of optimal thresholds. The upstaging, upgrading, and risk change rates observed in the cohort of 1056 cases were utilized to estimate the PPV and NPV. Odds ratios (OR) with 95% confidence intervals (CI) were calculated for combinations of biomarkers. *P* value of < 0.050 was considered significant.

## Results

### Overview of upgrading, upstaging, and risk change rates

**Fig. 1:**
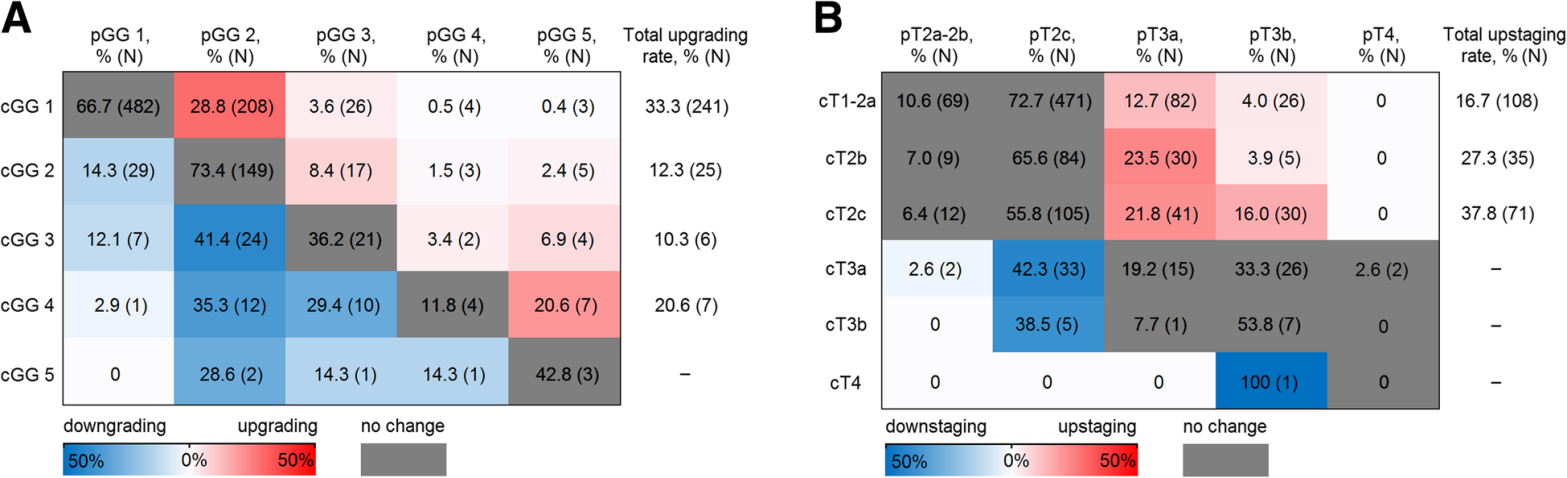
ISUP grade group (GG) or tumour stage (T) change rates after pathological examination of surgical material. **a** Upgrading/downgrading rates; **b** upstaging/ downstaging rates. Rates of GG and T change are accompanied with colours, where more intense blue depicts higher rate of downgrading/downstaging, while more intense red depicts higher rate of upgrading/upstaging. For visual purposes, the colour scale is capped at 50%

In order to gain a more accurate picture of PCa misclassification rates, clinico-pathological data of 1056 patients were analysed (Additional file 1: Table S1). Upgrading was observed in 27.2% (279/1025) of the patients, while 20.3% (214/1056) of the patients upstaged post-RP. The total misclassification rate, defined as the change of at least one of the two parameters, i.e. upgrading and/or upstaging, was 39.0% (400/1025). Among the upgraded cases, 86.4% (241/279) of the patients were initially diagnosed with cGG 1 disease, the majority of whom (86.3%, 208/241) were upgraded to pGG 2 disease (Fig. 1a). Patients initially diagnosed with the cT1c cancer dominated among the upstaged cases (49.1%; 105/214; Fig. 1b).

Histopathological examination of the whole prostate gland after RP revealed that 23.9% (245/1025) of the patients were assigned to a higher postoperative PCa risk group than clinically suspected, of whom 69.8% (*N* = 171) had been preoperatively diagnosed with low-risk PCa. Upgrading alone was the major cause of the risk increase (45.3%, *N* = 111), whereas both GG and tumour stage increase were identified in 22.5% (*N* = 55) and only stage increase—in 32.2% (N = 79) of the cases. The majority of the patients underwent RP within 3.8 ± 0.2 months after biopsy, excluding the potential possibility of PCa progression to adverse surgical pathology.

### DNA methylation of the selected TSGs

**Fig. 2:**
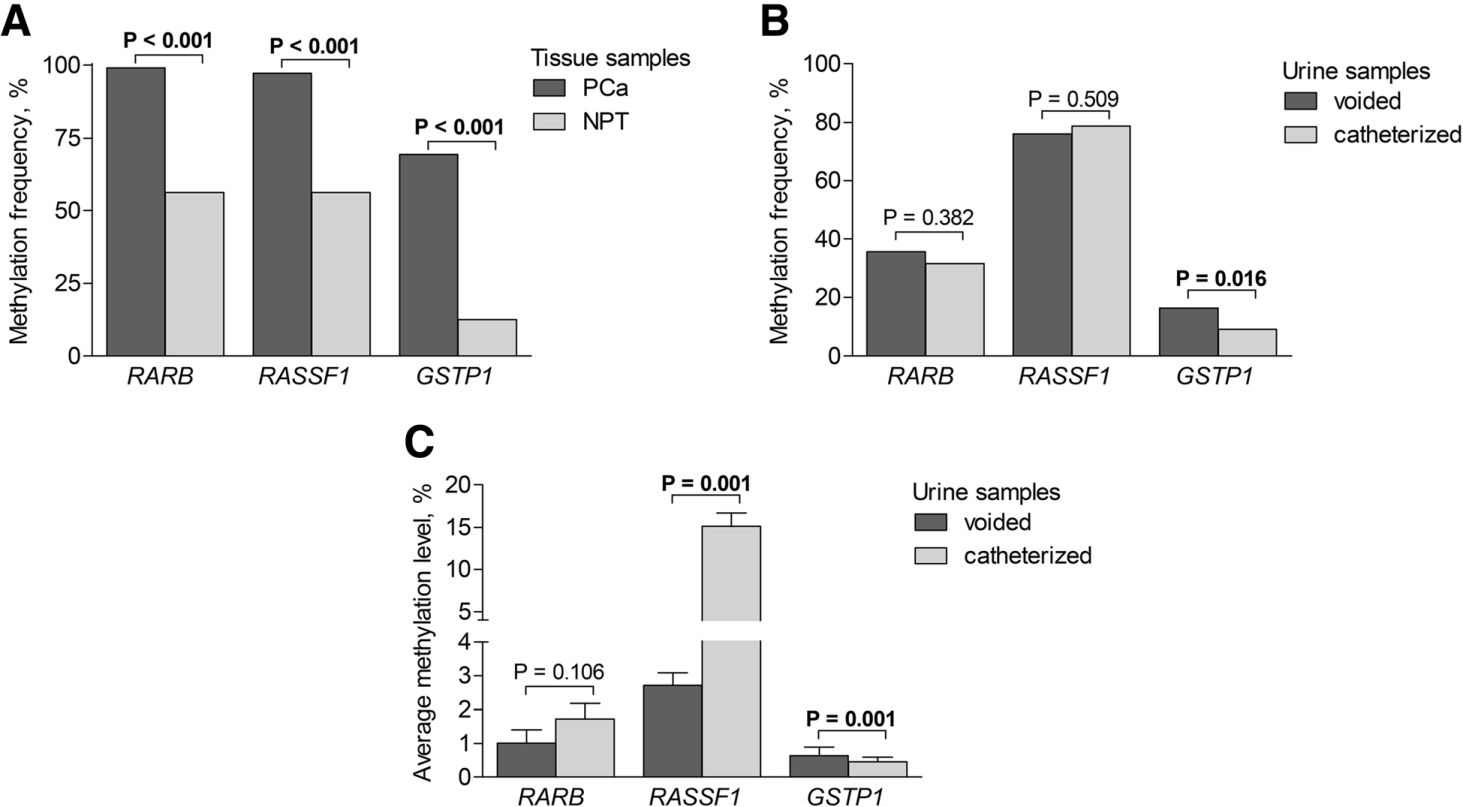
DNA methylation of genes *RARB*, *RASSF1*, and *GSTP1* in tissue and urine samples of patients diagnosed with prostate cancer. **a** Methylation frequencies in prostate tumours (PCa) and noncancerous prostate tissues (NPT); **b** methylation frequencies in urine samples; **c** average methylation levels of the genes in urine samples (± SEM). Significant *P* values are in bold.

DNA methylation of three TSGs—*RARB*, *RASSF1*, and *GSTP1*—was evaluated for the potential to improve PCa risk assessment before RP. Before analysing in urine, methylation status of the three-gene panel was first validated in prostatic tissues. Differently from NPT, the high frequency of promoter DNA methylation detected by qualitative MSP was observed in prostate tumours (all *P* < 0.001; Fig. 2a). In urine samples, methylation frequencies were similar between the voided and catheterized urine cohorts, except for *GSTP1* (*P* = 0.016; Fig. 2b). Overall, methylation of at least one gene of the three-gene panel was detected in 80.3% (151/188) of voided and 83.7% (273/326) of catheterized urine. The average methylation levels ranged from 0.6 to 15.1% and were significantly different for *RASSF1* and *GSTP1* between the voided and catheterized urine samples (both *P* = 0.001; Fig. 2c). This could be partially explained by different compositions of the two cohorts according to the clinico-pathological characteristics (Additional file 1: Table S1). Therefore, the voided and catheterized urine samples were further analysed as separate cohorts.

### Urinary DNA methylation as a biomarker of PCa upstaging and upgrading

We further evaluated the three-gene (*RARB*, *RASSF1*, and *GSTP1*) and two-gene (*RASSF1* and *GSTP1*) methylation as potential noninvasive biomarkers of PCa upstaging and upgrading.

**Fig. 3:**
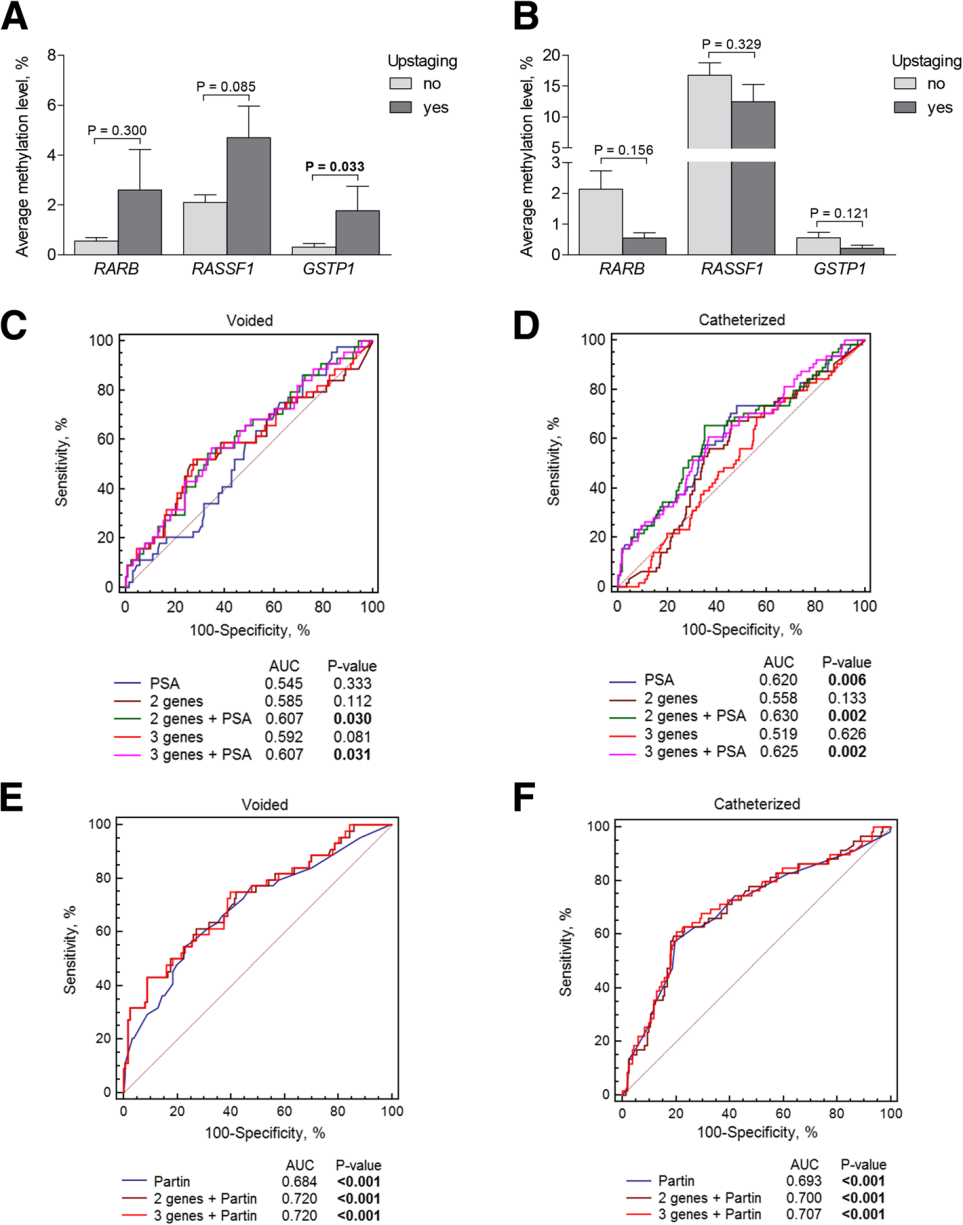
DNA methylation of *RARB*, *RASSF1*, and *GSTP1* as a biomarker of upstaging. **a** Methylation levels according to tumour stage change in voided urine samples; **b** methylation levels according to tumour stage change in catheterized urine samples; **c** comparison of ROC curves of the three-gene methylation in voided urine samples alone and combined with PSA; **d** comparison of ROC curves of the gene methylation in catheterized urine samples alone and combined with PSA; **e** comparison of ROC curves of the Partin value separately and in combination with the gene methylation in voided urine samples; **f** comparison of ROC curves of the Partin value separately and in combination with the gene methylation in catheterized urine samples. *RASSF1* and *GSTP1* together are referred to as the two genes. Significant *P* values are in bold

In voided urine, *GSTP1* showed significant differences in methylation level (*P* = 0.033) between cases with increased tumour stage and those with no upstaging; however, no associations were observed in the catheterized samples (Fig. 3a, b). In ROC analysis, methylation of each gene individually or in combinations was not an independent predictor for stage change in either cohort (all *P* > 0.050), while PSA level was predictive for upstaging in the catheterized urine cohort only. However, methylation of *RASSF1* or *GSTP1* alone or as the two-gene set, as well as the three-gene set, significantly complemented PSA in predicting higher postoperative pT in both urine cohorts (all *P* < 0.050; Table 1 and Fig. 3c, d). The NPV values for various biomarker combinations were around 85–86% and comparable to those of PSA (88–89%), whereas other test parameters had low-to-moderate values (Table 2).

**Table 1:** ROC curve values of particular gene methylation alone and combined with PSA as biomarkers of upstaging, upgrading, and risk increase

Gene	Voided urine (*N* = 188)				Catheterized urine (*N* = 326)			
	Gene methylation only		Gene methylation + PSA		Gene methylation only		Gene methylation + PSA	
	AUC	*P* value	AUC	*P* value	AUC	*P* value	AUC	*P* value
Upstaging								
*RARB*	0.551	0.330	0.596	0.053	0.554	0.154	0.626	**0.002**
*RASSF1*	0.586	0.106	0.608	**0.029**	0.540	0.313	0.632	**0.001**
*GSTP1*	0.575	0.062	0.608	**0.033**	0.533	0.174	0.627	**0.018**
Upgrading								
*RARB*	0.508	0.862	0.532	0.470	0.521	0.600	0.682	**< 0.001**
*RASSF1*	0.581	0.074	0.600	**0.025**	0.571	0.082	0.692	**< 0.001**
*GSTP1*	0.503	0.919	0.517	0.704	0.550	0.065	0.715	**< 0.001**
Risk increase								
*RARB*	0.558	0.194	0.518	0.692	0.510	0.816	0.589	**0.045**
*RASSF1*	0.538	0.434	0.537	0.426	0.523	0.599	0.597	**0.025**
*GSTP1*	0.539	0.189	0.521	0.634	0.557	**0.047**	0.623	**0.004**

Significant *P* values are in bold

**Table 2:** Sensitivity, specificity, and positive and negative predictive values (PPV and NPV) of the DNA methylation biomarkers (*RARB*, *RASSF1*, and *GSTP1*) and PSA for predicting upstaging, upgrading, and risk group increase

Parameter	Upstaging				Upgrading				Risk change			
	Sensitivity, %	Specificity, %	PPV, %	NPV, %	Sensitivity, %	Specificity, %	PPV, %	NPV, %	Sensitivity, %	Specificity, %	PPV, %	NPV, %
Voided urine (*N* = 188)												
PSA	86.7	27.9	23.4	89.1	82.6	27.0	29.7	80.6	85.0	27.4	26.9	85.3
*RARB*, *RASSF1*, *GSTP1*	52.3	72.5	32.6	85.6	50.7	67.5	36.9	78.6	45.8	71.3	33.4	80.7
*RARB*, *RASSF1*, *GSTP1*+PSA	56.8	65.2	29.4	85.6	47.8	74.6	41.2	79.3	89.8	27.0	27.9	89.4
*RASSF1*, *GSTP1*	50.0	73.9	32.8	85.3	50.7	69.3	35.7	78.1	45.8	71.3	33.4	80.7
*RASSF1*, *GSTP1+*PSA	54.5	66.7	27.7	84.6	40.3	81.6	45.0	78.5	89.8	27.0	27.9	89.4
Catheterized urine (*N* = 326)												
PSA	73.1	51.4	27.7	88.2	66.1	64.8	41.2	87.3	58.2	62.6	32.8	82.7
*RARB*, *RASSF1*, *GSTP1*	68.7	43.7	23.7	84.6	63.8	54.8	34.5	80.2	30.2	82.4	35.0	79.0
*RARB*, *RASSF1*, *GSTP1*+PSA	60.9	63.3	29.7	86.4	56,9	79,2	50.5	83.1	56.6	62.1	31.2	81.4
*RASSF1*, *GSTP1*	67.2	53.9	27.1	86.6	84.5	3.6	24.7	38.4	35.8	75.8	31.7	79.0
*RASSF1*, *GSTP1+*PSA	65.6	64.9	27.5	86.5	55.2	80.5	51.4	82.8	58.5	61.2	32.2	82.4

The biomarker performance in predicting upstaging was further evaluated together with the probability values according to Partin nomogram, which provides the chance of locally advanced disease. The Partin value was a significant predictor in both voided and catheterized urine cohorts, whereas the combination of the three-gene or the two-gene set only slightly increased the test performance, with more apparent difference observed in voided urine (Fig. 3e, f). In both cohorts, the addition of the three-gene set to Partin value increased the test’s sensitivity and NPV (Table 3).

**Table 3:** Sensitivity, specificity, positive and negative predictive values (PPV and NPV) of the DNA methylation biomarkers (*RARB*, *RASSF1* and *GSTP1*) together with Partin nomogram values for predicting upstaging

Parameter	Sensitivity, %	Specificity, %	PPV, %	NPV, %
Voided urine (*N* = 188)				
Partin	53.3	77.0	37.1	86.6
*RARB*, *RASSF1*, *GSTP1*+Partin	75.0	60.3	32.5	90.5
*RASSF1*, *GSTP1*+Partin	43.2	91.3	55.7	86.3
Catheterized urine (*N* = 326)				
Partin	56.5	79.8	41.6	82.4
*RARB*, *RASSF1*, *GSTP1*+Partin	61.0	79.8	43.5	88.9
*RASSF1*, *GSTP1*+Partin	59.3	80.8	44.0	88.6

**Fig. 4:**
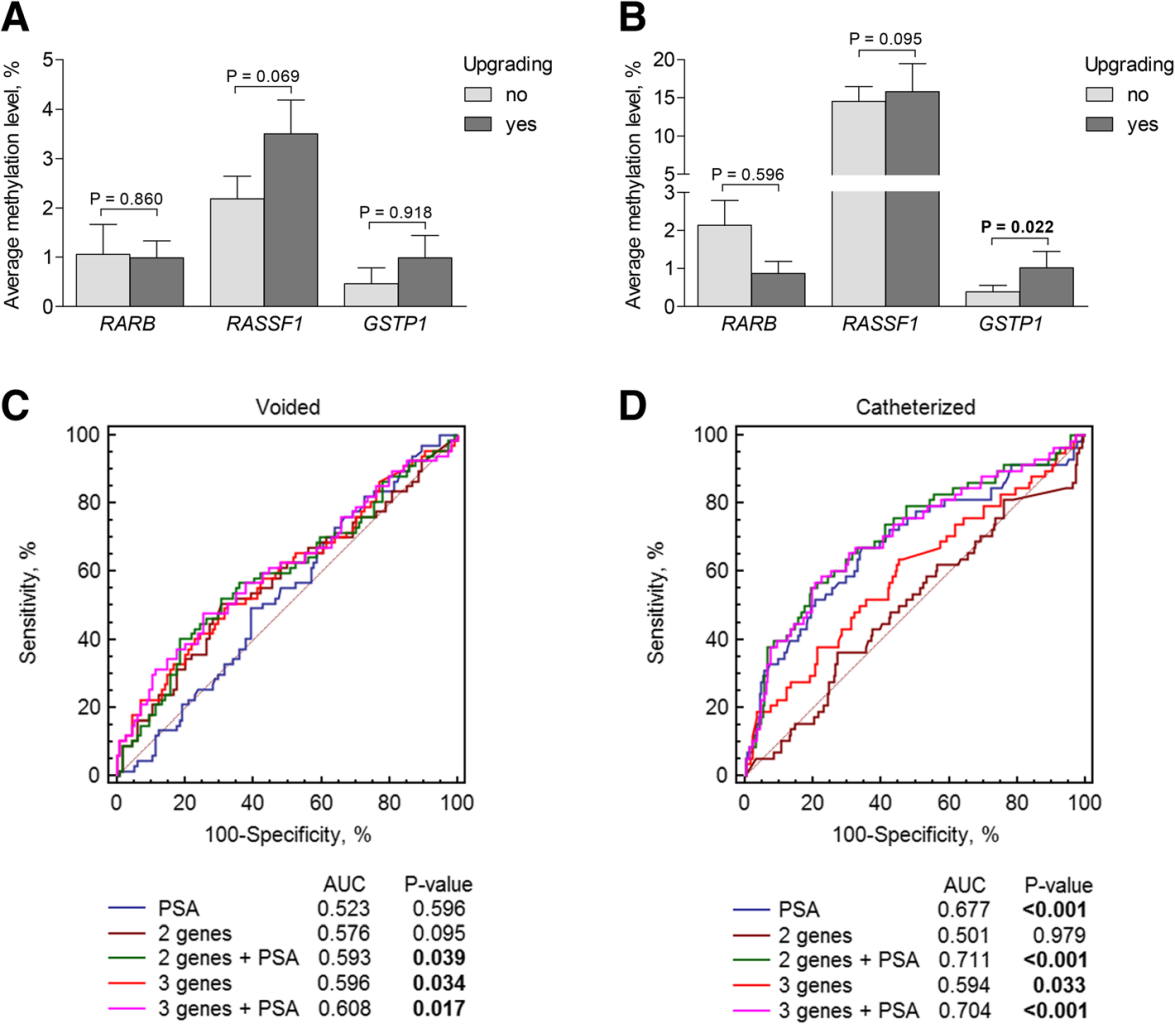
DNA methylation of *RARB*, *RASSF1*, and *GSTP1* as a biomarker of upgrading. **a** Methylation levels according to ISUP group change in voided urine samples; **b** methylation levels according to ISUP group change in catheterized urine samples; **c** comparison of ROC curves of the gene methylation levels in voided urine samples alone and combined with PSA; **d** comparison of ROC curves of the gene methylation levels in catheterized urine samples alone and combined with PSA. *RASSF1* and *GSTP1* together are referred to as the two genes. Significant *P* values are in bold

In the catheterized urine cohort, higher levels of *GSTP1* methylation were detected in the cases with postoperative upgrading (*P* = 0.022), while no association was detected in the voided samples (Fig. 4a, b). Similarly to upstaging analysis, PSA level was informative for the GG change in the catheterized urine cohort only (Fig. 4c, d). Meanwhile, the three-gene or the two-gene panels together with PSA test, as well as the three-gene panel separately, were predictive for upgrading (all *P* < 0.050) in both catheterized and voided urine cohorts (Fig. 4c, d). However, the biomarker performance parameters did not exceed those of the PSA test (Table 2).

The three-gene methylation together with PSA showed the highest odds ratios for tumour stage and GG change. According to this model, the odds for upstaging and upgrading were 122.2 [2.3–6556.6] and 111.5 [3.0–4077.9] in voided urine and 169.1 [8.6–3335.0] and 213.0 [13.6–3339.4] in catheterized urine (Additional file 2: Table S2).

### Urinary DNA methylation as a biomarker of PCa risk assessment

**Fig. 5:**
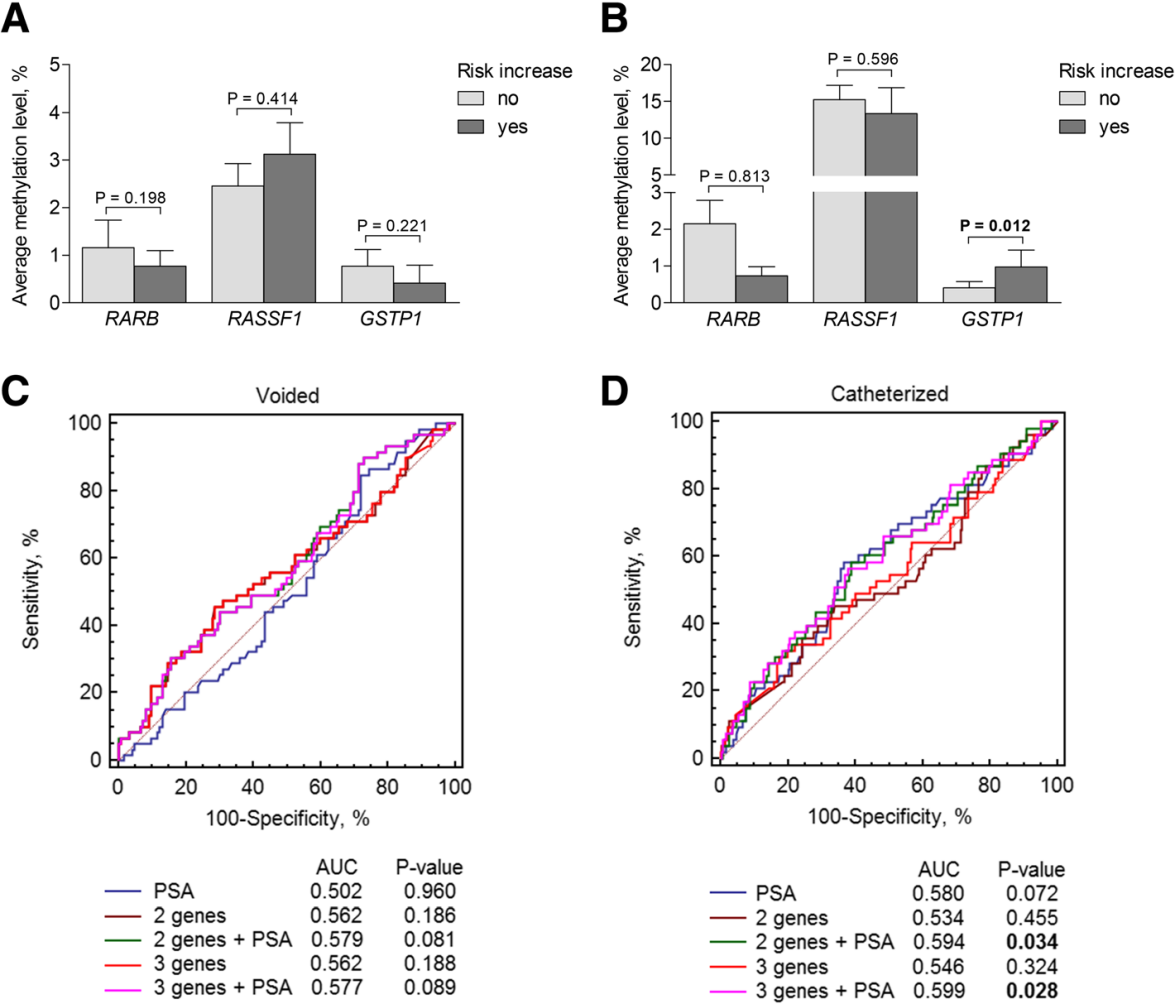
DNA methylation of *RARB*, *RASSF1*, and *GSTP1* as biomarker of risk increase. **a** Methylation levels according to risk increase in voided urine samples; **b** methylation levels according to risk increase in catheterized urine samples; **c** comparison of ROC curves of the gene methylation levels in voided urine samples alone and combined with PSA; **d** comparison of ROC curves of the gene methylation levels in catheterized urine samples alone and combined with PSA. *RASSF1* and *GSTP1* together are referred to as the two genes. Significant *P* values are in bold

The gene methylation biomarkers were further evaluated for the potential to predict the general PCa risk change after pathological examination. Methylation levels of *GSTP1* in the catheterized urine were higher in cases with the increased risk after RP (*P* = 0.012; Fig. 5a, b). ROC analysis revealed that the PSA test alone was not informative for the prediction of the increased PCa risk in either cohort; however, together with the three-gene or the two-gene test, it was able to predict the risk change when the catheterized urine was used and showed a weak tendency in the voided urine samples (Fig. 5c, d). Besides, all the genes individually complemented the prognostic power of PSA in the catheterized urine (all *P* < 0.050; Table 1). However, the test parameters were again similar among all the biomarker combinations (Table 2). In voided and catheterized urine cohorts, patients were 83.5 and 280.4 times more likely to have risk group increase according to the three-gene methylation when combined with PSA, respectively (Additional file 2: Table S2).

## Discussion

The optimal management of PCa patients is critically dependent on the accurate disease characterization at diagnosis. Currently used risk stratification models are mostly based on the original D’Amico system, however, with questionable accuracy [14, 15]. High upgrading (42%) and upstaging (29%) rates have been reported after RP, with corresponding association with inferior cancer-specific survival [1–3]. According to our data, 27.2% of PCa patients experienced upgrading and 20.3% upstaging after RP, providing the total misclassification rate as high as 39.0%.

The most common problems causing such discrepancy between initial prostate biopsy and RP material are mainly attributable to sampling and analysis errors. A sampling error could occur when a higher GS is missed on the needle biopsy. Vice versa, almost 20% of RP specimens have a tertiary Gleason grade which can be captured in biopsy but missed during histopathological examination of RP material, especially when partial embedding technique is considered [16, 17]. Besides, borderline neoplastic changes can be interpreted differently by different pathologists, especially when high quality samples are unavailable [16, 18]. Previous studies [19] have demonstrated that extended prostate biopsy is associated with less upgrading; however, it is not an issue in the current era when extended biopsy is the standard of care.

Rapid development of genomic technologies, so greater understanding of molecular carcinogenesis, has opened new diagnostic possibilities for PCa patients. It is becoming increasingly evident that particular epigenetic alterations appear to be nearly universal in a given cancer type, including PCa. The highly recurrent nature of these alterations can be exploited for biomarkers development for cancer detection and risk stratification [20]. To date, more than 100 DNA methylation biomarkers have been investigated for the potential to improve PCa diagnostics and prognostics, while only few of them have proven clinical value [9]. Tissue-based epigenetic alterations of *RASSF1*, *GSTP1*, and *APC* urine-based testing of *PCA3* or *TMPRSS2-ERG* together with *PCA3* have been proposed for improved PCa diagnostics. All these novel tests are used in combination with the PSA test and have a potential to reduce the number of unnecessary biopsies, but have a limited potential to predict PCa aggressiveness [11, 21, 22]. Following our previous study [6], linking *RASSF1* methylation with PCa aggressiveness, and showing a good diagnostic power of the 3-gene methylation test, we tested the diagnostic potential of this noninvasive tool in the settings of the PCa risk change after RP.

In the present study, the performance of *RARB*, *RASSF1*, and *GSTP1* methylation, as biomarkers for upstaging, upgrading, and risk change, was analysed in two independent cohorts with different urine collection techniques, i.e. post-prostatic massage voided and catheterized during RP urine samples. The general *GSTP1* methylation level, as well as frequency, was relatively low, although significantly different between the cohorts. More intense *GSTP1* methylation was observed in voided urine of patients who underwent upstaging, whereas it was associated with upgrading and risk group change only in catheterized samples. Altogether, this is in agreement with numerous studies reporting associations between *GSTP1* methylation and aggressiveness of the disease [23]. Slightly higher *RASSF1* methylation levels were observed in upgraded cases from both cohorts; however, only weak tendencies were detected, while *RARB* did not show potential in predicting clinical parameter changes. Our study revealed a limited value of each gene individually for predicting post-RP upstaging and upgrading; however, in combination with PSA, all three genes (or *RASSF1* and *GSTP1* only) revealed moderate test performance parameters in both voided and catheterized urine cohorts. The combination of PSA together with the epigenetic biomarkers was also predictive of the risk group change, but only in the catheterized urine cohort. The discrepancies observed between the two cohorts could be explained by the different gene methylation levels detected in voided and catheterized urine, especially those of *RASSF1* and *GSTP1*. This was most likely related to the different techniques employed for urine sample collection, which should be taken into consideration when interpreting epigenetic data. Some previous studies [4] suggested that higher PSA level and older patient age could be associated with upgrading and upstaging, and this observation was partially supported by our data. Nevertheless, the highest AUC values have been reached in PSA combinations with the gene methylation biomarkers, supporting the added value and, thus, clinical significance of the three-gene test in PCa assessment. However, considering the wide confidence intervals of OR values, further investigations in the large independent groups of PCa cases are beneficial to prove the prognostic power of this test in predicting the risk change at diagnosis.

In the last decade, multiparametric magnetic resonance imaging (mpMRI) has emerged as a promising diagnostic modality. Despite low overall sensitivity (47%), mpMRI has proved good sensitivity for GS ≥ 7 disease, although the detection rate is significantly influenced by tumour volume which is frequently underestimated [24–26]. The negative predictive value of MRI decreases with the increasing prevalence [27], so it is necessary to risk-stratify the patients for whom clinically significant PCa could be ruled out safely when mpMRI is negative. Current studies [28] have shown that mpMRI-ultrasound fusion biopsies are associated with less upgrading; however, the design of our study was initiated when pelvic mpMRI was rarely used for diagnostic purposes and the results of mpMRI were available only for a small number of patients, thus were not taken into consideration.

To the best of our knowledge, this is the first study investigating a urine-based epigenetic test in combination with the serum PSA for predicting pathological upstaging/upgrading after RP. Nevertheless, we must address several limitations of the present study. Firstly, some differences in clinico-pathological characteristics between the patients in voided and catheterized urine cohorts were identified, which could have influenced the differences observed in the methylation levels and frequencies. Secondly, although the clinical data were maintained prospectively, the analysis was performed in a retrospective way. Furthermore, no pathological re-evaluation of biopsy specimens from outside institutions were performed so inter-observer variation might affect the results. Finally, mpMRI, widely used in nowadays clinical practice, was not included in our protocol. Notwithstanding these limitations, the study revealed the clinical value of the three well-known epigenetic biomarkers for more precise PCa assessment by comparing two large, well-characterized cohorts of voided and catheterized urine samples.

## Conclusions

Currently available diagnostic tools do not allow precise preoperative PCa risk assessment, especially in the low-risk group where a significant subset of a higher risk disease is missed. According to the literature and our data, a total misclassification rate is close to 40%. Our study revealed that combination of the urinary three-gene test and serum PSA may improve individual PCa risk assessment at the point of treatment selection. However, the clinical value of such combined testing is somewhat limited and requires future studies in independent cohorts, which might reveal a true potential of these biomarkers for predicting PCa risk change after RP.

## Additional files

Additional file 1: **Table S1.** Clinico-pathological characteristics of all cohorts. (DOCX 31 kb)

Additional file 2: Odds ratios of the gene methylation and PSA for predicting upstaging, upgrading, and risk group change. (DOCX 17 kb)

## Data Availability

All data supporting the results reported in the article is available from the corresponding author upon a reasonable request.

## Abbreviations

*APC*: Adenomatous polyposis coli gene
AUC: Area under the curve
cGG: Clinical ISUP grade group
CI: Confidence interval
cT: Clinical tumour stage
DNA: Deoxyribonucleic acid
GG: ISUP grade group
GS: Gleason score
*GSTP1*: Glutathione *S*-transferase pi 1 gene
ISUP: International Society of Urological Pathology
MC: *In vitro* methylated control
mpMRI: Multiparametric magnetic resonance imaging
MSP: Methylation-specific polymerase chain reaction
NPT: Noncancerous prostate tissues
NPV: Negative predictive value
NTC: Nontemplate control
OR: Odds ratio
PCa: Prostate cancer
*PCA3*: Prostate cancer-associated 3
PCR: Polymerase chain reaction
pGG: Pathological ISUP grade group
PPV: Positive predictive value
PSA: Prostate-specific antigen
pT: Pathological tumour stage
*RARB*: Retinoic acid receptor β gene
*RASSF1*: RAS association domain family member 1 gene
ROC: Receiver operating characteristic
RP: Radical prostatectomy
SEM: Standard error of mean
*TMPRSS2-ERG*: The transmembrane protease serine 2 and v-ets erythroblastosis virus E26 oncogene homolog gene fusion
TSG: Tumour suppressor gene
