# Supplementary material for: Urinary DNA methylation biomarkers for prediction of prostate cancer upgrading and upstaging
Source: Clin Epigenetics. 2019 Aug 5;11:115. doi: 10.1186/s13148-019-0716-z (PMC6683454; doi:10.1186/s13148-019-0716-z)
Supplement: Supplementary file 1 — Table S1. Clinico-pathological characteristics of all cohorts. (DOCX 31 kb) [file 13148_2019_716_MOESM1_ESM.docx]

**Table S1.** Clinico-pathological characteristics of all cohorts

| **Parameter** | **All cases (N = 1056)** | **Tumor tissues (N = 111)** | **Catheterized urine (N = 326)** | **Voided urine (N = 188)** | **P-value*** |
| --- | --- | --- | --- | --- | --- |
| Age, years^a^ | | | | | |
| Mean (±SEM) | 62.2 (0.2) | 60.6 (0.8) | 61.7 (0.4) | 62.2 (0.6) | 0.382 |
| Median | 63 | 61 | 62 | 62 |  |
| PSA, ng/mL^b^ | | | | | |
| Mean (±SEM) | 8.4 (0.3) | 10.3 (1.0) | 8.6 (0.5) | 8.4 (0.7) | 0.613 |
| Median | 6.0 | 6.7 | 6.2 | 5.8 |  |
| cGG, n (%)^c^ | | | | | |
| 1 | 723 (70.5) | 69 (64.5) | 226 (81.0) | 93 (51.4) | **<0.001** |
| 2 | 203 (19.8) | 21 (19.6) | 42 (15.0) | 55 (30.4) |  |
| 3 | 58 (5.7) | 9 (8.4) | 7 (2.5) | 24 (13.2) |  |
| 4 | 34 (3.3) | 6 (5.6) | 3 (1.1) | 7 (3.9) |  |
| 5 | 7 (0.7) | 2 (1.9) | 1 (0.4) | 2 (1.1) |  |
| pGG, n (%)^a^ | | | | | |
| 1 | 539 (51.0) | 38 (34.2) | 207 (67.0) | 39 (21.4) | **<0.001** |
| 2 | 405 (38.4) | 50 (45.1) | 86 (27.8) | 108 (59.3) |  |
| 3 | 76 (7.2) | 16 (14.4) | 13 (4.2) | 28 (15.4) |  |
| 4 | 14 (1.3) | 2 (1.8) | 1 (0.3) | 1 (0.6) |  |
| 5 | 22 (2.1) | 5 (4.5) | 2 (0.7) | 6 (3.3) |  |
| Upgrading, n (%)^c^ | | | | | |
| Yes | 279 (27.2) | 39 (36.4) | 58 (20.8) | 67 (37.0) | **<0.001** |
| No | 746 (72.8) | 68 (63.6) | 221 (79.2) | 114 (63.0) |  |
| cT stage, n (%)^a^ | | | | | |
| ≤cT1c | 637 (60.3) | 59 (53.2) | 189 (61.2) | 112 (61.5) | 0.597 |
| cT2a | 11 (1.1) | 2 (1.8) | 4 (1.3) | 3 (1.7) |  |
| cT2b | 128 (12.1) | 17 (15.3) | 28 (9.0) | 30 (16.5) |  |
| cT2c | 188 (17.8) | 20 (18.0) | 73 (23.6) | 25 (13.7) |  |
| cT3a | 78 (7.4) | 13 (11.7) | 12 (3.9) | 10 (5.5) |  |
| cT3b | 13 (1.2) | - | 3 (1.0) | 2 (1.1) |  |
| cT4 | 1 (0.1) | - | - | - |  |
| pT stage, n (%)^a^ | | | | | |
| pT2a | 80 (7.6) | 4 (3.6) | 36 (11.7) | 3 (1.7) | **0.006** |
| pT2b | 12 (1.1) | - | 1 (0.3) | 3 (1.7) |  |
| pT2c | 698 (66.1) | 63 (56.8) | 199 (64.4) | 122 (67.0) |  |
| pT3a | 169 (16.0) | 30 (27.0) | 48 (15.5) | 37 (20.3) |  |
| pT3b | 95 (9.0) | 13 (11.7) | 24 (7.8) | 17 (9.3) |  |
| pT4 | 2 (0.2) | 1 (0.9) | 1 (0.3) | - |  |
| Upstaging, n (%)^a^ | | | | | |
| Yes | 214 (20.3) | 32 (28.8) | 64 (20.7) | 44 (24.2) | 0.371 |
| No | 842 (79.7) | 79 (71.2) | 245 (79.3) | 138 (75.8) |  |
| Preoperative PCa risk, n (%)^c^ | | | | | |
| Low | 501 (48.9) | 44 (41.1) | 152 (54.5) | 70 (38.7) | 0.104 |
| Intermediate | 243 (23.7) | 29 (27.1) | 47 (16.8) | 71 (39.2) |  |
| High | 281 (27.4) | 34 (31.8) | 80 (28.7) | 40 (22.1) |  |
| Postoperative PCa risk, n (%)^a^ | | | | | |
| Low | 489 (46.3) | 35 (31.5) | 183 (59.2) | 38 (20.9) | **<0.001** |
| Intermediate | 290 (27.5) | 30 (27.0) | 52 (16.8) | 88 (48.3) |  |
| High | 277 (26.2) | 46 (41.5) | 74 (24.0) | 56 (30.8) |  |
| Risk increase, n (%)^c^ | | | | | |
| Yes | 245 (23.9) | 31 (29.0) | 53 (19.0) | 59 (32.6) | **0.001** |
| No | 780 (76.1) | 76 (71.0) | 226 (81.0) | 122 (67.4) |  |
| Partin value, %^d^ | | | | | |
| Mean (±SEM) | 27.2 (0.4) | 29.3 (1.3) | 26.3 (0.8) | 29.1 (1.0) | **0.024** |
| Median | 27 | 28 | 25 | 29 |  |

Abbreviations: cGG = clinical ISUP grading; cT = clinical T-staging; ISUP = International Society for Urological Pathology; pGG = pathological ISUP grading; pT = pathological T-staging; PCa = prostate cancer; PSA = prostate-specific antigen; SEM = standard error of mean; T = local tumour staging according to TNM classification.
^a^ Age, pISUP grade, cT-stage, pT-stage, stage change and postoperative PCa risk missing in 17 patients of catheterized urine cohort, and in 6 patients of voided urine cohort.
^b^ PSA missing in 10 patients of all cohort, in 25 patients of catheterized urine cohort, and in 6 patients of voided urine cohort.
^c^ cISUP grade, preoperative PCa risk, grade change and risk change missing in 31 patients of all cohort, in 4 patients of tumour tissue cohort, in 47 patients of catheterized urine cohort, and in 7 patients of voided urine cohort.

^d^ Partin value missing in 145 patients of all cohort, in 15 patients of tumour tissue cohort, in 63 patients of catheterized urine cohort, and in 17 patients of voided urine cohort.
^*^ P-values calculated for comparison of catheterized and voided urine cohorts only.
