# Supplementary material for: Urinary DNA methylation biomarkers for prediction of prostate cancer upgrading and upstaging
Source: Clin Epigenetics. 2019 Aug 5;11:115. doi: 10.1186/s13148-019-0716-z (PMC6683454; doi:10.1186/s13148-019-0716-z)
Supplement: Supplementary file 2 — Odds ratios of the gene methylation and PSA for predicting upstaging, upgrading, and risk group change. (DOCX 17 kb) [file 13148_2019_716_MOESM2_ESM.docx]

**Table S2.** Odds ratios of the gene methylation and PSA for predicting upstaging, upgrading, and risk group change.

| Gene | Voided urine (N = 188) | | | Catheterized urine (N = 326) | | |
| --- | --- | --- | --- | --- | --- | --- |
|  | Odds ratio | CI | P-value | Odds ratio | CI | P-value |
| Upstaging | | | | | | |
| PSA | 1.0 | [1.0; 1.0] | 0.857 | 1.1 | [1.0; 1.1] | **<0.001** |
| *RARB*, *RASSF1*, *GSTP1* | 120.1 | [2.2; 6545.0] | **0.009** | 1742.6 | [0.1; >10000] | 0.078 |
| *RARB*, *RASSF1*, *GSTP1* + PSA | 122.2 | [2.3; 6556.6] | **0.008** | 169.1 | [8.6; 3335.0] | **<0.001** |
| *RASSF1*, *GSTP1* | 113.1 | [2.2; 5717.0] | **0.009** | 585.6 | [<0.1; >10000] | 0.252 |
| *RASSF1*, *GSTP1* + PSA | 115.2 | [2.3; 5742.5] | **0.008** | 143.0 | [7.4; 2773.5] | **<0.001** |
| Upgrading | | | | | | |
| PSA | 1.0 | [0.9; 1.0] | 0.385 | 1.1 | [1.0; 1.1] | **<0.001** |
| *RARB*, *RASSF1*, *GSTP1* | 110.6 | [2.8; 4422.2] | **0.005** | 199.7 | [1.2; 32676.8] | **0.040** |
| *RARB*, *RASSF1*, *GSTP1* + PSA | 111.5 | [3.0; 4077.9] | **0.004** | 213.0 | [13.6; 3339.4] | **<0.001** |
| *RASSF1*, *GSTP1* | 69.9 | [0.4; >10000] | 0.092 | 68.7 | [0.1; 35643.5] | 0.192 |
| *RASSF1*, *GSTP1* + PSA | 69.7 | [0.6; 8442.0] | 0.075 | 171.0 | [10.4; 2801.1] | **<0.001** |
| Risk increase | | | | | | |
| PSA | 1.0 | [0.9; 1.0] | 0.401 | 1.0 | [1.0; 1.1] | 0.130 |
| *RARB*, *RASSF1*, *GSTP1* | 84.5 | [0.8; 9115.3] | 0.059 | 416.0 | [1.4; >10000] | **0.037** |
| *RARB*, *RASSF1*, *GSTP1* + PSA | 83.5 | [1.0; 7048.2] | **0.046** | 280.4 | [2.7; >10000] | **0.018** |
| *RASSF1*, *GSTP1* | 85.2 | [0.8; 9215.9] | **0.045** | 118.5 | [0.1; >10000] | 0.171 |
| *RASSF1*, *GSTP1* + PSA | 84.5 | [1.0; 7164.5] | **0.045** | 103.5 | [0.7; >10000] | 0.074 |

P-values of the logistic regression models are provided. CI – 95% confidence intervals.
